# Curcumin Provides Hepatoprotection against Amoebic Liver Abscess Induced by *Entamoeba histolytica* in Hamster: Involvement of Nrf2/HO-1 and NF-*κ*B/IL-1*β* Signaling Pathways

**DOI:** 10.1155/2019/7431652

**Published:** 2019-04-15

**Authors:** José Roberto Macías-Pérez, Liseth Rubí Aldaba-Muruato, Sandra Luz Martínez-Hernández, Martín Humberto Muñoz-Ortega, Julieta Pulido-Ortega, Javier Ventura-Juárez

**Affiliations:** ^1^Química Clínica, Unidad Académica Multidisciplinaria Zona Huasteca, Universidad Autónoma de San Luis Potosí, Ciudad Valles, SLP, Mexico; ^2^Departamento de Morfología, Centro de Ciencias Básicas, Universidad Autónoma de Aguascalientes, Aguascalientes, Ags, Mexico; ^3^Departamento de Química, Centro de Ciencias Básicas, Universidad Autónoma de Aguascalientes, Aguascalientes, Ags, Mexico

## Abstract

Amoebic liver abscess (ALA) is the most common extraintestinal amoebiasis caused by *Entamoeba histolytica* (*E. histolytica*). However, despite current knowledge and scientific advances about this infection, there are no effective treatments to prevent it. Herein, the antiamoebic capacity of curcumin in a hamster model was evaluated. Curcumin (150 mg/kg, p.o., daily during 10 days before infection) considerably prevents liver damage induced at 12 and 48 h post-intrahepatic inoculation of trophozoites and decreases ALT, ALP, and *γ*-GTP activities, and macroscopic and microscopic observations were consistent with these results. On the other hand, after one week of intraportal inoculation, liver damage was prevented by curcumin (150 mg/kg, p.o., daily, 20 days before amoebic inoculation and during the week of infection); liver/body weight ratios and tissue and histological stains showed normal appearance; in addition, the increases in ALT, ALP, and *γ*-GTP activities were prevented; the depletion of glycogen content induced by the amoebic damage was partially but significantly prevented, while NF-*κ*B activity was inhibited and the expression of IL-1*β* was reduced; Nrf2 production showed a tendency to increase it, and HO-1 protein was overexpressed. These results suggest for the first time that curcumin can be a compound with antiamoebic effect in the liver, suggesting that its daily use could help greatly decrease the incidence of this type of infection.

## 1. Introduction

Amoebiasis caused by the protozoan *Entamoeba histolytica* (*E. histolytica*) remains a public health problem with clinical importance because it has a worldwide distribution, being more frequent in low-income countries, such as México, Central and South America, India, and Africa [1–3]. *E. histolytica* primarily infects the colon; however, it can become invasive, taking advantage of its capacities for adherence, motility, and cytotoxicity towards host cells, migrating to the liver causing amoebic liver abscess (ALA), which leads to high morbidity and mortality. This pathology is characterized by the presence of one or multiple abscesses, as a result of uncontrolled inflammation, accompanied by the production of reactive oxygen species (ROS) [4]. It has been recently shown that hepatic invasion by *E. histolytica* increases the oxidative stress and proinflammatory cytokine production through the nuclear factor-kappa B (NF-*κ*B) activation and on the other hand inhibits the nuclear factor erythroid 2- (NF-E2-) related factor 2 (Nrf2) pathway, an important inducer of antioxidant enzymes, favoring the evolution of liver damage [5]. In the present work, we evaluated curcumin (diferuloylmethane) during amoebic liver infection in hamster because several authors have postulated that this bioactive component of turmeric (*Curcuma longa*) ameliorates cellular responses to oxidative stress through the activation of transcription factor Nrf2 [6, 7] as well as suppresses inflammation by inhibiting NF-*κ*B [8, 9]. Likewise, numerous reports have described that curcumin possesses antioxidant, anti-inflammatory, antifibrotic, antinecrotic, and anticancer properties [8, 10].

So far, there are no prophylactic treatments for amoebiasis. The prevention measures are based on hand hygiene, disinfection of water and vegetables, and avoiding street food and other fecal-oral exposure [11]. However, these strategies are sometimes difficult to carry out, mainly in developing countries or in environmental catastrophes. The present work proposes curcumin as an alternative to prevent hepatic amoebiasis by protecting the liver naturally.

## 2. Materials and Methods

### 2.1. Chemicals

Carboxymethylcellulose (CMC), anthrone, 4-nitroaniline, L-*γ*-glutamyl-p-nitroaniline, p-nitrophenyl phosphate, 4-nitrophenol, DL alanine, *α*-ketoglutaric acid, 2,4-dinitrophenylhydrazine, sodium pyruvate, and bovine serum albumin were purchased from Sigma Chemical Company (St. Louis, MO, USA). Sodium hydroxide, glacial acetic acid, hydrochloric acid, sulfuric acid, ethanol, methanol, potassium hydroxide, and formaldehyde were obtained from J.T. Backer (Xalostoc, Mexico). Curcumin (C7727, Sigma-Aldrich) with ≥94% curcuminoid content and ≥80% curcumin was also obtained. All the reagents were of analytical quality.

### 2.2. Animals

Male golden hamsters (*Mesocricetus auratus*) weighing 140-160 g were used in this study. The animals were maintained on standard diet with free access to drinking water. All animals received human care according to the guidelines of the Committee on Bioethics in the animal facilities of the Autonomous University of Aguascalientes, Aguascalientes, Mexico, which is based on the guidelines for animal research published by the National Institute of Health [12].

### 2.3. Intrahepatic Amoebic Infection

The trophozoites of the *E. histolytica* strain HM-1:IMSS were passed multiple times through animal livers to maintain virulence and were grown under axenic conditions in Diamond's TYI-S-33 medium at 36°C [13]. Trophozoites were harvested in logarithmic phase of growth. 5×10^5^ trophozoites were then inoculated into the left lobe of the hamster liver in a volume of 100 *μ*L of culture medium as previously described [14, 15]. The hepatic amoebiasis infection was carried out in animals anaesthetized with sodium pentobarbital (50 mg/kg, i.p.).

### 2.4. Intraportal Amoebic Infection

The abdominal cavity of each hamster was opened, the portal vein was exposed, and the inoculum of virulent trophozoites (5 × 10^5^) was slowly injected [14].

### 2.5. Evaluation of Hepatoprotective Effect of Curcumin on Early Stages of ALA Induced by Intrahepatic Infection

**Figure 1:**
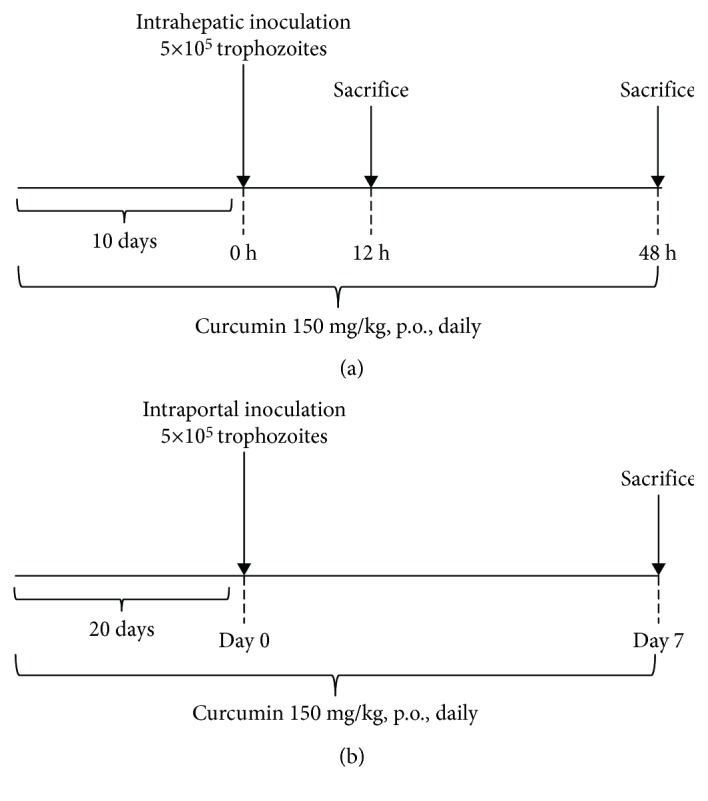
Experimental protocols of amoebic liver infection in hamster. (a) Intrahepatic inoculation: hamsters were pretreated 10 days with curcumin (150 mg/kg, p.o., daily) before intrahepatic infection (5 × 10^5^ trophozoites), until sacrifice; 5 animals were sacrificed at 12 h and 5 more at 48 h after amoebic infection. (b) Intraportal infection: the animals were pretreated 20 days with curcumin (150 mg/kg, p.o., daily) before intraportal infection (5 × 10^5^ trophozoites) and continued the same treatment until sacrifice.

Virulent trophozoites of *E. histolytica* were inoculated intrahepatically in hamsters (Figure 1(a)), and then the animals were sacrificed at 12 (ALA 12 h group, *n* = 5) and 48 h (ALA 48 h group, *n* = 5). In addition, control animals were included that underwent the same surgical procedure; however, the inoculum was Diamond's medium free of amoebas, and the sham hamsters were sacrificed at 12 and 48 h, respectively (sham 12 and sham 48 h groups, *n* = 5 in each one). To evaluate the effects of curcumin, 10 animals were pretreated with curcumin (150 mg/kg, p.o. daily for 10 days) before amoebic liver infection, and then 5 animals were sacrificed at 12 h (curcumin+ALA 12 h group, *n* = 5), and the other 5 animals were sacrificed at 48 h (curcumin+ALA 48 h group, *n* = 5). Additionally, sham groups treated with curcumin were included (curcumin 12 and 48 h groups, *n* = 5). Finally, 5 healthy animals were included as additional control (intact group). Curcumin was administered as suspension in 0.5% CMC at a final volume of 0.5 mL.

### 2.6. Evaluation of Hepatoprotective Effect of Curcumin at One-Week ALA Induced by Intraportal Infection

Virulent trophozoites of *E. histolytica* were inoculated intraportally in hamsters (ALA group, *n* = 5), and sham-operated animals inoculated with Diamond's medium free of amoebas were included (sham group, *n* = 5). To evaluate the effects of curcumin, 10 animals were pretreated with curcumin (150 mg/kg, p.o., daily for 20 days) before amoebic inoculation (curcumin+ALA group, *n* = 5) or sham surgery (curcumin group, *n* = 5). Both animal groups continued curcumin treatment until being sacrificed. Finally, 5 healthy animals were added as additional control (intact group). All animals were sacrificed 7 days after amoebic infection or sham surgery (Figure 1(b)). All animals were weighed before surgery and sacrifice.

### 2.7. Sacrificed Animals

We followed the methods of Aldaba-Muruato et al. [16]; these methods are briefly described. Animals were anaesthetized with sodium pentobarbital (50 mg/kg, i.p.). Blood was collected via cardiac puncture, and the livers were carefully dissected free from the surrounding fatty and fibrous tissues and immediately rinsed in saline solution 0.9%. Fragments of the liver were snap frozen in liquid nitrogen and stored at -20°C until use. Additional liver fragments were taken and fixed in 4% formaldehyde phosphate buffered saline. In the one-week amoebiasis study, the liver/body weight ratio was determined.

### 2.8. Biochemical Estimations

We followed the methods of Aldaba-Muruato et al. [16]; these methods are briefly described. Blood samples were collected and centrifuged at 3000 ×g for 20 min at 4°C. Serum was used for the determination of liver damage by measuring ALP [17], *γ*-GTP [18], and ALT activities [19]. Small liver pieces of amoebic abscess (0.1 g) were separated for determination of glycogen with the anthrone reagent [20].

### 2.9. Isolation of Total Proteins

TRI Reagent® (Sigma-Aldrich T9424) was used to isolate total protein from samples of liver tissue. Total protein was determined by Bradford's method [21].

### 2.10. Western Blot Assays

We followed the methods of Aldaba-Muruato et al. [16]; these methods are briefly described. Volumes equivalent to 30 *μ*g of total proteins were transferred on polyacrylamide gel 12%; separated proteins were transferred onto Immuno-Blot TM PVDF Membranes (Bio-Rad, 162-0.176, Hercules, CA, USA). Next, blots were blocked with 5% skimmed milk and 0.05% Tween-20 for 1 h at room temperature and independently incubated at room temperature with antibodies against each protein, phospho NF-*κ*B Ser536 (pNF-*κ*B, Cell Signaling 3033), Nrf2, HO-1 (LifeSpan BioSciences, LS-C154863, LS-C15743, respectively), and IL-1*β* (Millipore, MAB1001). Membranes were washed and then exposed to horseradish peroxide-conjugated anti-mouse, anti-goat, and anti-rabbit IgG (Sigma, A9044, A5420, A0545), respectively, diluted 1 : 2000 in blocking solution for 1 h at room temperature. Blots were washed and protein developed using the Clarity Western ECL Substrate (Bio-Rad, 170-5061). Blots were incubated with a monoclonal antibody directed against *β*-actin (Sigma, A2066), which was used as a control to normalize protein levels. The procedure to strip membranes was as follows: first, blots were washed four times with phosphate buffered saline pH 7.4 (0.015 M, 0.9% NaCl) and then immersed in stripping buffer (2-mercaptoethanol 100 mM, sodium dodecyl sulfate 2% and Tris–HCl 62.5 mM, pH 6.7) for 30 min at 60°C with gentle shaking; membranes were then washed five times with 0.05% Tween-20 in phosphate buffered saline. The protein expressions were analyzed densitometrically using the ImageJ software.

### 2.11. Hematoxylin and Eosin Staining

Liver samples were taken from all the animals and fixed with 4% formaldehyde in phosphate buffered saline for 24 h. Tissue pieces were washed with tap water, dehydrated in alcohol, and embedded in paraffin. Five-micrometer sections were mounted on silane-treated glass slide. Hematoxylin and eosin staining (H&E) was performed.

### 2.12. Statistical Analysis

Data are expressed as mean values ± SE. Comparisons were carried out by analysis of variance followed by Tukey's test, as appropriate, using GraphPad Prism 5.00 software. Differences were considered statistically significant when *p* < 0.05.

## 3. Results

### 3.1. Evaluation of Hepatoprotective Effect of Curcumin on Early Stages of ALA Induced by Intrahepatic Infection

**Figure 2:**
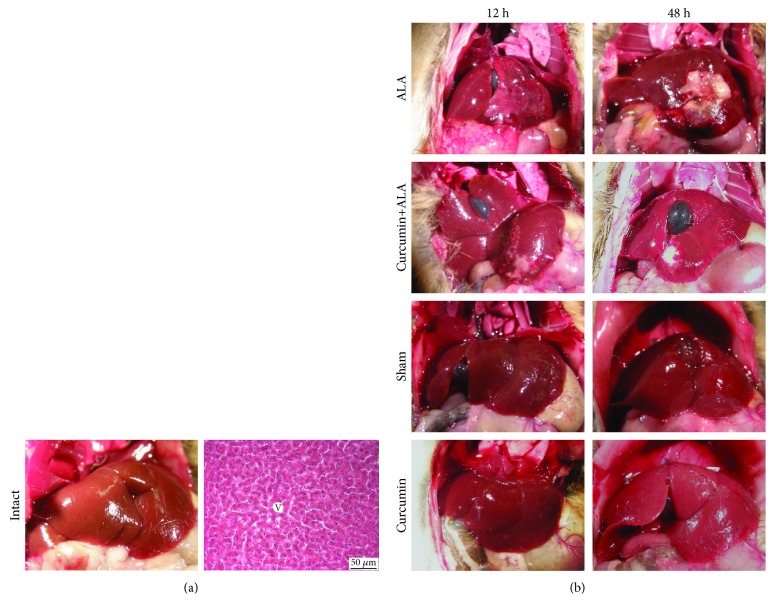
Hepatoprotective activity of curcumin during early stage of liver infection of *Entamoeba histolytica*. Representative images of the groups. (a) Intact: macroscopic and microscopic observations. The hematoxylin and eosin stain (H&E) shows a normal architecture. V: central vein. Scale bar = 50 *μ*m. (b) Macroscopic analysis at 12 and 48 h: ALA, curcumin+ALA, sham, and curcumin.

**Figure 3:**
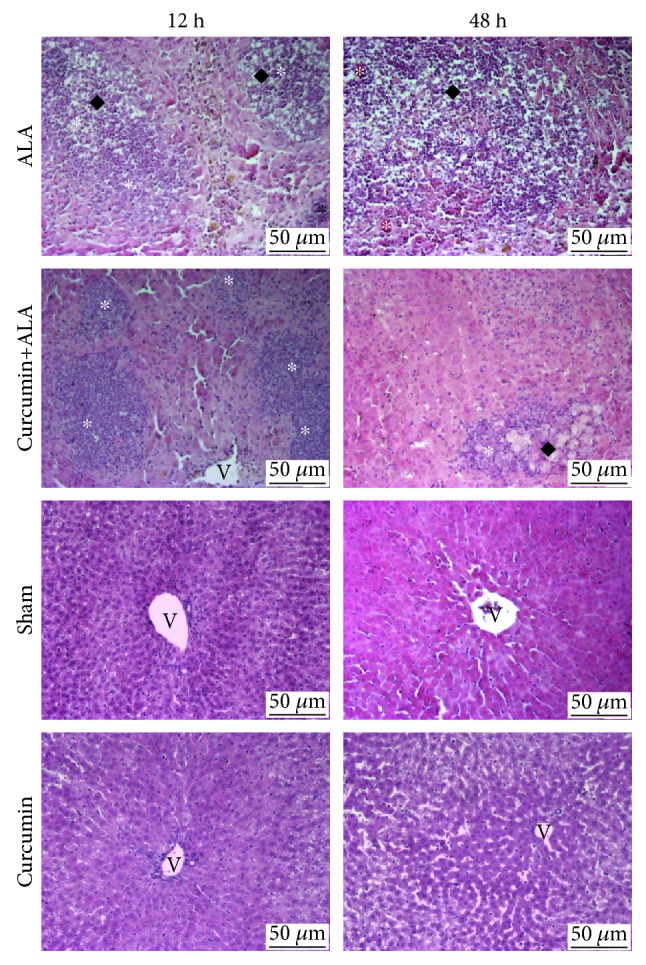
Microscopic observations to evaluate the hepatoprotective activity of curcumin during early stages of infection of *Entamoeba histolytica*. Representative images of H&E of the groups at 12 h and 48 h: intact; ALA, curcumin+ALA, sham, and curcumin. Presence of granulomas with central area of necrosis (black diamond) and inflammatory infiltrate (^∗^). V: central vein. Scale bar = 50 *μ*m.

Macroscopic observations of livers (Figure 2) suggest a hepatic damage induced by amoebas at 12 h, as well as greater damage at 48 h. On the other hand, the pretreatment with curcumin partially prevented ALA development at 12 h, presenting greater protection at 48 h. The healthy controls at 12 and 48 h (intact, sham, and curcumin groups) presented a normal appearance with uniform color. On the other hand, microscopic analysis of the healthy controls (intact, sham, and curcumin groups) at 12 and 48 h clearly showed the normal structure of the hepatocytes (Figures 2(a) and 3). At 12 h post infection, small granulomas with necrotic centers and inflammatory cells were observed, surrounded by morphologically unaltered hepatocytes, and at 48 h, larger areas of granulomatous inflammation with central areas of necrosis were observed. The pretreatment with curcumin reduced the presence of granulomas, finding at 12 h small abscesses with inflammatory content, surrounded with normal hepatic parenchyma, while at 48 h smaller granulomas were seen, and most of the tissue was constituted of healthy cells with normal architecture, although a scattered inflammatory cell infiltrate was observed near granulomas (Figure 3).

**Figure 4:**
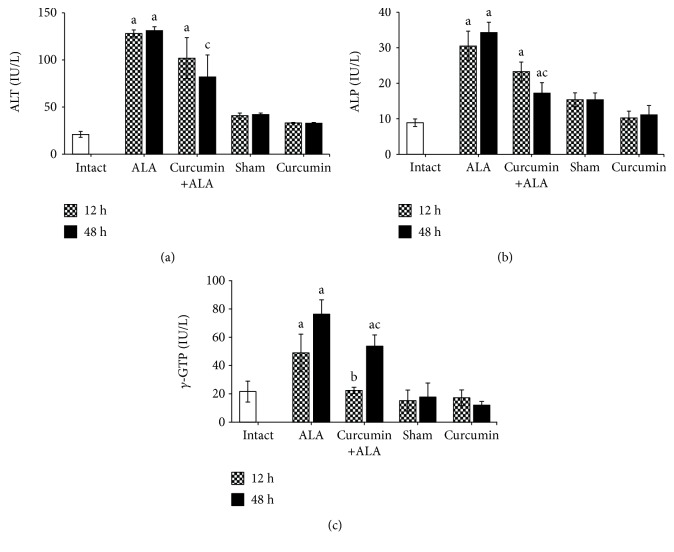
Enzymatic activities of markers of liver damage in serum samples. (a) Alanine aminotransferase (ALT), (b) alkaline phosphatase (ALP), (c) *γ*-glutamyl transpeptidase (*γ*-GTP). Intact group (white bars) and infected with trophozoites during 12 h (checkered bars) and 48 h (black bars). Each bar represents the mean value ± SE, *n* = 5 tested by duplicated assays. a: mean values significantly different from the intact group, *p* < 0.05; b: mean values significantly different from the sham group at 12 h, *p* < 0.05; c: mean values significantly different from the sham group at 48 h, *p* < 0.05.

Serum markers of liver damage were tested for the animals infected with *E. histolytica* at 12 and 48 h; the ALT, ALP, and *γ*-GTP activities increased significantly with respect to the healthy groups (intact, sham, and curcumin). Conversely, after 12 h of infection, curcumin showed tendencies to decrease ALT and ALP activities and significantly prevented the increase of *γ*-GTP, while at 48 h post infection the increase in the activities of the three markers of liver damage was smaller than in the ALA group (Figure 4).

### 3.2. Evaluation of Hepatoprotective Effect of Curcumin on One-Week ALA Induced by Intraportal Infection

**Figure 5:**
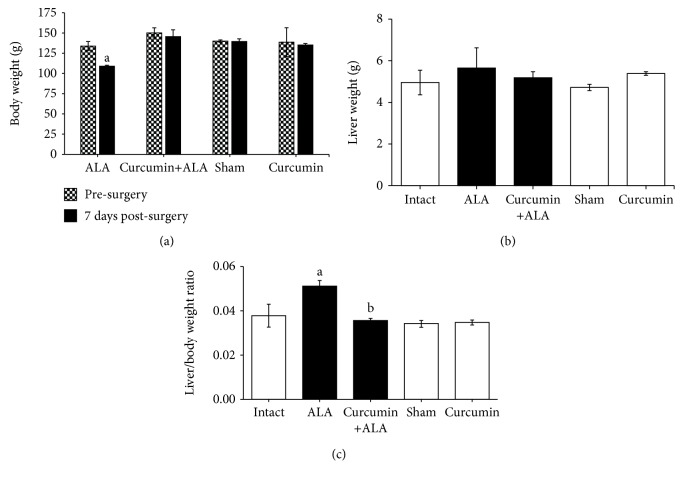
Changes in body and liver weight on the one-week study of ALA induced by intraportal inoculation of *E. histolytica*. (a) Average body weight of ALA, curcumin+ALA, sham, and curcumin groups before surgery (checkered bars) and after 7 days of surgery (black bars). a: significant difference with respect to the values obtained before intraportal infection. (b) Average liver weights of each group. (c) Ratio of liver weight to body weight. (b, c) Healthy groups (white bars) and infected animals (black bars). a: significant difference with respect to healthy groups (intact, sham, and curcumin); b: significant difference with respect to the ALA group (*p* < 0.05).

After seven days of intraportal infection, the ALA group significantly lose weight compared with the presurgery body weight (Figure 5(a)), while the weights of the livers show a tendency to increase (Figure 5(b)), and liver/body ratio was significantly increased compared to healthy controls (Figure 5(c)). On the other hand, the pretreatment with curcumin prevents these alterations.

**Figure 6:**
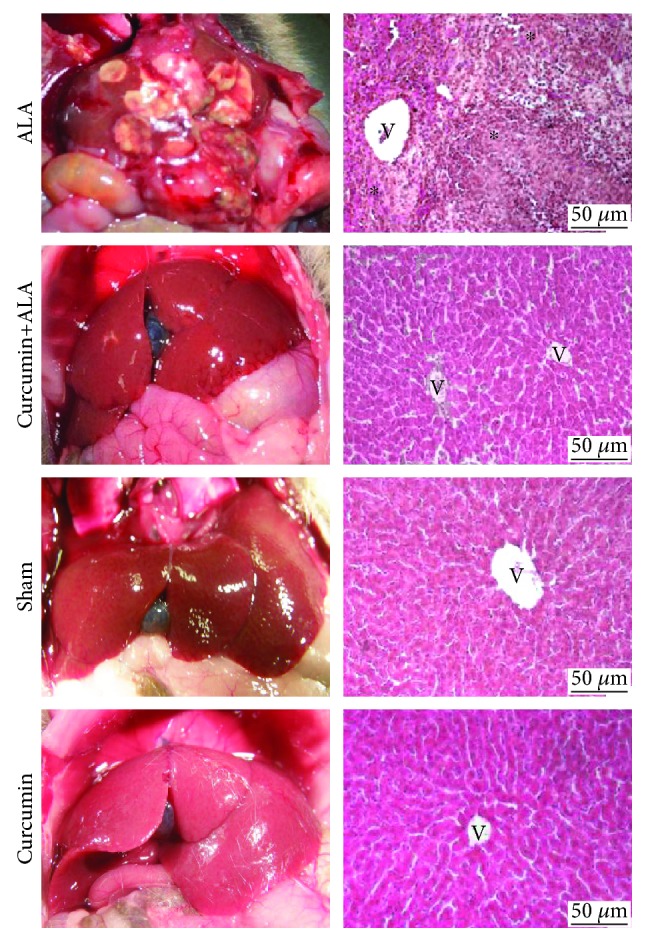
Hepatoprotective activity of curcumin evaluated after 7 days of liver infection induced by *Entamoeba histolytica*. Representative images of macroscopic and microscopic (H&E) observations of ALA, curcumin+ALA, sham, and curcumin groups. (^∗^) Presence of granulomas with central area of necrosis and inflammatory content. V: central vein. Scale bar = 50 *μ*m.

Moreover, 7-day postamoebic intraportal infection showed generalized liver damage, accompanied by adherence and apparent spread of damage to the surrounding tissues; H&E stain showed granulomas with the presence of necrosis and inflammatory cells coalescing to form large abscesses. Curcumin treatment prevents liver damage induced by *E. histolytica*; H&E stain showed a normal appearance with uniform staining in comparison with healthy animals (Figure 6).

**Figure 7:**
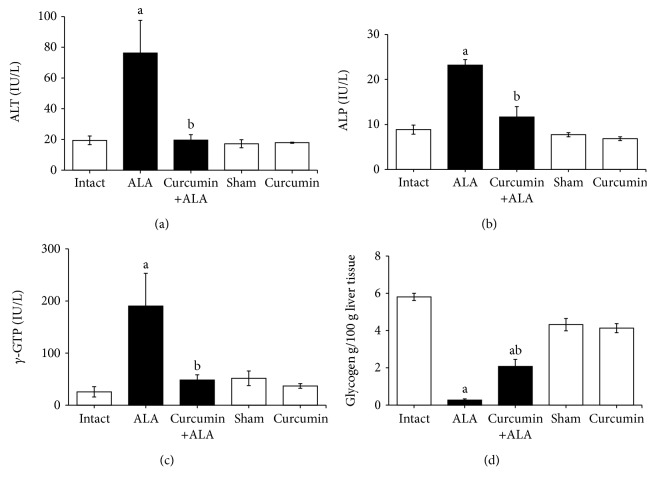
Evaluation of liver damage markers. Enzymatic activities in serum samples: (a) ALT, (b) ALP, and (c) *γ*-GTP; and in liver samples (d) glycogen content. Healthy animals (white bars) and infected with trophozoites (black bars). Each bar represents the mean value ± SE, *n* = 5; experiments were performed by duplicate. a: mean values significantly different from the intact group, *p* < 0.05; b: mean values significantly different from the ALA group, *p* < 0.05.

Serum activities of ALT, ALP, and *γ*-GTP from the ALA group were significantly increased with respect to the healthy groups (intact, sham, and curcumin). Conversely, after 7 days of infection, curcumin completely prevents these increases (Figures 7(a)–7(c)). In addition, glycogen content was reduced in the ALA group compared to the intact group, and the curcumin treatment partially prevented this depletion (Figure 7(d)).

**Figure 8:**
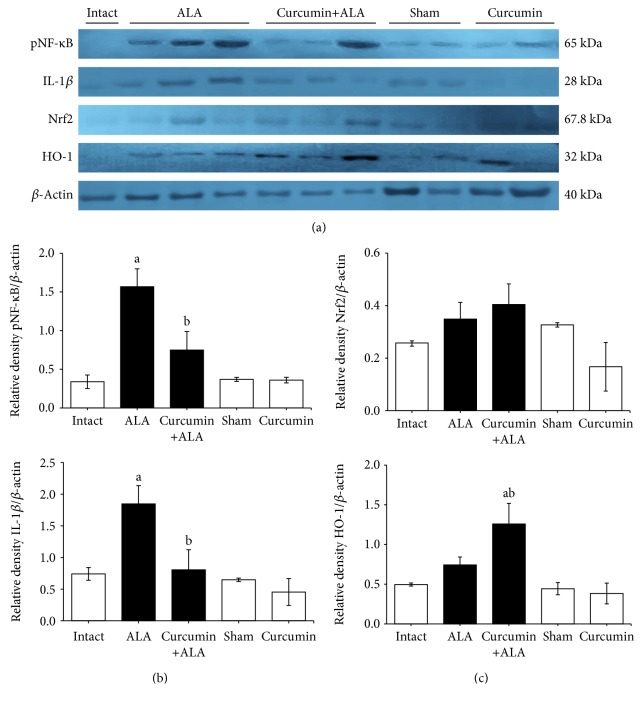
Protein expression of Nrf2 and NF-*κ*B. (a) Representative western blot of pNF-*κ*B, IL-1B, Nrf2, and HO-1 of liver samples of healthy controls (white bars) and infected hamsters (black bars). (b, c) Signal intensities were determined by densitometric analysis of the blots and values calculated as the ratio of each protein to *β*-actin. Results are shown as the mean value ± SE of 5 hamsters analyzed by three replicates. a: mean values significantly different from the intact group; b: mean values significantly different from the ALA group; (*p* < 0.05).

In the ALA group, the protein expressions of pNF-*κ*B and IL-1*β* were significantly increased in relation to the intact group (Figures 8(a) and 8(b)), whereas Nrf2 and HO-1 remained apparently unchanged (Figures 8(a) and 8(c)). Moreover, curcumin treatment significantly prevented the activation of NF-*κ*B and reduced the IL-1*β* expression (Figures 8(a) and 8(b)). On the other hand, although the Nrf2 expression did not show significant differences between groups, it was observed that curcumin increased the HO-1 expression (Figures 8(a) and 8(c)).

## 4. Discussion

ALA is a parasitic liver disease, secondary to intestinal infection by *E. histolytica*. In humans, ALA is usually reported as a single, large, and well-defined lesion; nevertheless, multiple abscesses are also frequent. These abscesses show necrotic areas with a creamy appearance and its aspirate is classically described as anchovy paste [22]. Histopathological observations reveal that the chronic phase of ALA in humans corresponds to lytic or liquefactive necrosis, whereas in rodent models there are necrosis and granulomatous inflammation [23]. In the present work, we induced ALA in hamsters, evidencing macroscopically and microscopically the amoebic liver abscess formation at 12 h and 48 h post-intrahepatic inoculation (Figures 2 and 3), likewise 7 days post-intraportal amoebic infection (Figure 6). The intrahepatic amoebic infection showed the formation of granulomas with necrotic centers containing inflammatory cells at 12 h, whereas at 48 h the granulomas tended to look larger. In addition, 7 days of amoebic damage induced extensive necrosis and inflammatory spread. Inflammatory process during ALA was fundamental to demonstrate the efficiency of curcumin treatment against hepatic amoebiasis because this compound is credited with great anti-inflammatory capacity. Our results showed that curcumin treatment significantly reduced the acute liver damage (12 h and 48 h post-intrahepatic infection) caused by *E. histolytica* (Figures 2 and 3) and completely prevented liver damage induced during 7 days by this parasite (Figure 6), given that less inflammatory area and better preservation of the lobular structure were observed.

On the other hand, hepatomegaly occurs in most patients with ALA [24]. Our results show that hamsters infected during 7 days with *E. histolytica* have increased liver/body weight ratio, suggesting the presence of hepatomegaly, and curcumin treatment prevented this alteration (Figure 5(c)).

Furthermore, the enzymatic determinations of ALT, ALP, and *γ*-GTP were carried out in order to evaluate the hepatic damage from experimental groups. Serum level of ALT is a clinical marker used to evaluate the presence of abnormal liver function [25]; this assay is considered the more specific marker for hepatic necrosis because ALT is normally found in the cytoplasm of the hepatocytes, whereas raised serum levels of ALP and *γ*-GTP are considered indicators of cholestasis because these enzymes are present on hepatocyte canalicular domain and the luminal domain of the bile duct epithelium [26]. Therefore, the increase of these three enzymes in serum suggests hepatic necrosis and damage to the biliary tract as a result of cytotoxic and cytolytic activity of the parasite. Our study shows that all animals infected with virulent amoebas (12 h, 48 h, and 7 days post infection) have increased ALT, ALP, and *γ*-GTP activities in serum (Figures 4 and 7). These biochemical results were consistent with a previous study of ALA in hamster [16]. Curcumin treatment shows hepatoprotective activity induced by *E. histolytica*, decreasing serum activities of ALT, ALP, and *γ*-GTP, which were consistent with macroscopic and microscopic observations, suggesting that curcumin protects in both the early and late stages of liver infection.

Moreover, glycogen is the main energy source in the body, involved in maintaining blood sugar homeostasis, and is an indicator of metabolism and liver functionality [20, 27]. Glycogen content was markedly reduced after 7 days of amoebic infection, while curcumin partially prevented this depletion, suggesting that the metabolic capacity of the liver is being preserved (Figure 7(d)).

Several reports suggest that curcumin protects the liver against hepatotoxic compounds, such as carbon tetrachloride, alcohol, and paracetamol [28–30]. Furthermore, antibacterial, antiviral, and antifungal activity of curcumin has been reported [31]. Likewise, curcumin has been shown to possess antiparasitic activity against malaria and *Giardia lamblia* [32, 33]. Recently, it was determined by trypan blue exclusion test that curcumin affects the growth and cell viability of *E. histolytica*, in a time- and dose-dependent manner [34]. In the present work, we studied the antiamoebic activity of curcumin in the ALA model in hamster, and our results suggest that curcumin could help to greatly decrease the incidence of this type of infection.

On the other hand, NF-*κ*B is an inducible transcription factor that regulates various cellular functions ranging from the inflammatory response to cell proliferation [35] and also directs gene expression in response to pathogens including *E. histolytica* [36]. Our western blot results showed significant increases in the active form of NF-*κ*B, as well as the expression of IL-1*β* in the ALA group, suggesting that during amoebiasis the parasite is able to stimulate the inflammatory process. Moreover, our results show that curcumin prevented the activation of NF-*κ*B, and therefore, this inhibition was consistent with the reduced expression of IL-1*β* (Figure 8). Other works are consistent with our results, which describe that curcumin exerts its beneficial effect by suppressing the activation of NF-*κ*B and reducing the expression of proinflammatory cytokines such as IL-1*β* and TNF-*α* [8]. Recently, it has been described that during the acute stage of amoebic infection NF-*κ*B signaling is activated, whereas the activation of factor Nrf2 is blocked and thus the expression of HO-1 is reduced [26]. Our results showed that in one-week amoebiasis the expressions of Nrf2 and HO-1 did not change significantly compared to healthy controls; however, curcumin induced the overexpression of HO-1 suggesting that Nrf2 is activated. Other studies have demonstrated that the activation of Nrf2/HO-1 signal pathway protects the liver from damage induced by alcohol or carbon tetrachloride [37, 38]. Few studies evaluate the modulation of the transcriptional factor Nrf2 during parasitic diseases, such as *Trypanosoma cruzi* infection in mice, where NRF2/HO-1 pathway was induced with cobalt protoporphyrin, which reduced parasitemia and tissue parasitism, while an inhibitor of HO-1 activity increased *T. cruzi* parasitemia [39]. It has been reported that curcumin attenuates hepatic damage induced by lipopolysaccharide/d-galactosamine by activating the nuclear translocation of Nrf2 and inhibiting the activation of NF-*κ*B [40]. Curcumin is an effective chemopreventive agent against oxidative and nitrative stress derived from praziquantel treatment during *Opisthorchis viverrini* infection via induction of Nrf2 and suppression of NF-*κ*B-mediated pathway [41].

In the present work, we demonstrated that the dose of 150 mg/kg of curcumin is effective to prevent amoebiasis in the hamster model. Therefore, these results suggested that it could be estimated for humans, a dose of 10.5 g of curcumin in young people and adults (70 kg), although it is important to consider that there are low weight averages across populations of the world, the reason why the dosages can be based on size and weight of the patient. In addition, curcumin is a compound with median lethal dose (LD50) higher than 1000 mg/kg in rats, eliciting some effects like initial excitement, followed by mild depression, dullness, decreased respiration, and reduced spontaneous motor activity [42].

## 5. Conclusion

Our results report for the first time that curcumin has an important hepatoprotective effect against infection induced by *E. histolytica* in hamster, possibly influenced by its antioxidant activity, coupled with its ability to activate the signaling of the antioxidant pathway of Nrf2, and its capacity to inhibit the activation of the transcription factor NF-*κ*B, suggesting for the first time that this compound may be a natural alternative to prevent the liver damage induced by this parasite. However, it is necessary to continue evaluating this compound due to its potential as an antiamoebic drug.

## Data Availability

The data that support the findings of this study are available from the corresponding author, JVJ, upon reasonable request.
